# *In vitro* and *in vivo* investigation for optimization of niosomal ability for sustainment and bioavailability enhancement of diltiazem after nasal administration

**DOI:** 10.1080/10717544.2016.1259371

**Published:** 2017-02-06

**Authors:** H. O. Ammar, M. Haider, M. Ibrahim, N. M. El Hoffy

**Affiliations:** 1 Department of Pharmaceutics and Pharmaceutical Technology, Faculty of Pharmaceutical Sciences and Pharmaceutical Industries, Future University in Egypt, Cairo, Egypt,; 2 Sharjah Institute for Medical Research, Department of Pharmaceutics and Pharmaceutical Technology, College of Pharmacy, University of Sharjah, Sharjah, UAE, and; 3 Department of Pharmaceutics and Industrial Pharmacy, Faculty of Pharmacy, Cairo University, Giza, Egypt

**Keywords:** Niosomes, diltiazem hydrochloride, nonionic surfactants, nasal delivery, pharmacokinetic parameters

## Abstract

Diltiazem hydrochloride (DTZ) is a calcium channel antagonist depicted by extensive first pass metabolism and low oral bioavailability. The aim of this work was to develop niosomes for potential nasal delivery of DTZ. Niosomes protect hydrophilic drugs inside their core while nasal route offers both rapid onset and evasion of first-pass metabolism. Niosomes were prepared using a combination of Span 60 or Brij-52 with cholesterol (CHOL) in different molar ratios followed by determination of entrapment efficiency, particle size and *in vitro* drug release. A parallel design was adopted to evaluate the pharmacokinetic performance of DTZ-loaded niosomes in male Wistar rats. Non-compartmental analysis was performed where C_max_, *T*_max_, *t*_1/2_, MRT, area under the release curve (AUC) and K_e_ were assessed. The prepared niosomes were spherical with mean particle size 0.82–1.59 μm. Span 60-cholesterol niosomes (1:1 molar ratio) showed the highest entrapment and release efficiencies. *In vivo* study revealed an increase in MRT, *t*_1/2_ and AUC with a decrease in K_e_. In conclusion, nasal niosomal formulation of DTZ expressed suitable pharmacokinetic parameters and bioavailability through prolonged duration of action inside the body as well as low rate of elimination depicting a promising alternate to the conventional oral route.

## Introduction

Diltiazem hydrochloride (DTZ) is a benzothiazepine calcium channel antagonist indicated for treatment of hypertension, angina pectoris as well as some types of arrhythmia (Zhang et al., 2010). The mean plasma half-life of DTZ is 3–6 h and usually 80% of a dose is rapidly absorbed from the gastrointestinal tract after oral administration. However, oral bioavailability is only 30–60% due to its extensive hepatic first-pass metabolism primarily via cytochrome P450 enzymes, mainly CYP3A4 (Echizen & Eichelbaum, 1986; Boyd et al., 1989; Höglund & Nilsson, 1989; Pinto et al., 2005). Intravenous administration of DTZ is not frequently recommended partly due to lack of patients’ compliance and also due to intense irritation. This creates a need for alternative routes of administration for DTZ to improve its bioavailability and therapeutic efficacy (Kulkarni et al., 2016).

Recent investigations suggested the use of intranasal route as an alternative to oral and parenteral administrations for systemic delivery of various therapeutic compounds that are susceptible to acidic or enzymatic degradation and hepatic first-pass metabolism (Hussain, 1998; Pontiroli, 1998; Romeo et al., 1998; Illum, 2000,2003; Song et al., 2004). The large surface area of the nasal mucosa offers a rapid onset of action due to direct drainage from nose to systemic circulation associated with no first pass metabolism which not only results in an increase in bioavailability of the drug but also leads to dose reduction and minimization of possible side effects. In addition, nasal delivery is noninvasive which may maximize convenience, comfort, and compliance of the patient. However, short residence time of therapeutic agents in nasal cavity after nasal application due to mucociliary clearance mechanism (Belgamwar et al., 2011; Chalikwar et al., 2013), obstruction of airflow, congestion and decongestion nasal cycle and sensitivity of nasal mucosa are among the factors affecting the drug permeation and systemic bioavailability via the nasal route which limits the use of this route of administration in drug delivery (Illum, 2003). Accordingly, many studies have investigated different techniques to increase nasal membrane permeability such as using permeation enhancers (Piao et al., 2010), polymeric mucoadhesive nanoparticles (Ugwoke et al., 2005), liposomes (Qiang et al., 2012) and other carriers which can encapsulate an active drug and enhance its release and absorption across the nasal membrane. The physicochemical and structural properties of these systems were varied to maximize the stability, absorption and therapeutic efficacy and reduce toxicity of the active ingredient (Dhakar et al., 2011).

Niosomes are bilayered vesicles of a combination of nonionic surfactants and CHOL or its derivatives. This characteristic structural arrangement allows for possible co-encapsulation of hydrophilic and lipophilic substances where the first is entrapped in the vesicular aqueous core or adsorbed on the surface of the bilayer while the later can be embedded in the lipophilic domain. Niosomes are biodegradable, non-immunogenic and less toxic than their corresponding micellar vesicles composed of cationic, amphoteric and anionic surfactants due to their nonionic nature (Kazi et al., 2010). Niosomes are also superior to other micro and nanoparticulate carriers such as liposomes due to the higher stability of nonionic surfactants when compared to phospholipid molecules used in liposome formulations which results in longer shelf-life and less susceptibility to photodegradation (Mahale et al., 2012). In addition, niosomes require simple methods for manufacturing and large-scale production which increases their cost-effectiveness. Niosomes were initially used in formulation of cosmetic products (Moghassemi & Hadjizadeh, 2014); however, many recent studies emphasized the potential of niosomes as carriers for controlled delivery of drugs (Shahiwala & Misra, 2002; Junyaprasert et al., 2012; Bendas et al., 2013) proteins (Arunothayanun et al., 1999; Pardakhty et al., 2007), oligonucleotides (Huang et al., 2008) and vaccines (Vyas et al., 2005; Jain et al., 2005) via intravenous (Leroux et al., 1995; Hong et al., 2009; Waddad et al., 2013), intramuscular (Vyas et al., 2005), oral (Leroux et al., 1995; Gurrapu et al., 2012; Abdallah et al., 2013), ocular (Abdelkader et al., 2011; Abdelkader et al., 2012), pulmonary (Marianecci et al., 2010; Elhissi et al., 2013), intraperitoneal (Walker et al., 1996) and transdermal (Manosroi et al., 2010; Ammar et al., 2011; Junyaprasert et al., 2012) routes of administration.

In this work, nonionic surfactant-based niosomes encapsulating DTZ were developed for nasal administration. The effect of varying the type of nonionic surfactant used as well as the molar concentration of CHOL incorporated into the lipid bilayer on the physicochemical properties and *in vitro* release of the drug were studied. Moreover, based on the *in vitro* results, selected niosomes formulae were evaluated *in vivo* by measuring blood concentration of DTZ after nasal administration in rats. To the best of our knowledge, this is the first reported study on using niosomes for nasal delivery of DTZ.

## Materials and methods

### Materials

DTZ was obtained as a kind gift from EIPICO Co. (10th of Ramadan, Egypt). CHOL was purchased from Bio Basic Inc. (Ontario, Canada). Sorbitan monostearate (Span 60), polyethylene glycol hexadecyl ether (Brij52), acetonitrile (HPLC grade) and triethanolamine (HPLC grade) and methanol were acquired from Sigma-Aldrich (St. Louis, MO). Chloroform, disodium hydrogen orthophosphate anhydrous (Min Assay-acidimetric 98%), sodium dihydrogen orthophosphate-1-hydrate (Min Assay 98%), orthophosphoric acid and ether (Min Assay 99% GC) were obtained from ADWIC (Qalyubia, Egypt).

### Preparation of DTZ niosomes

DTZ niosomes were prepared using Thin Film Hydration method (Agarwal et al., 2001). Briefly, 400 mg of nonionic surfactants (Span 60 or Brij 5) and CHOL were mixed at different molar concentrations (Table 1) and dissolved in 20 mL chloroform/methanol mixture in the ratio of 2:1 (v/v) in a round bottom flask. A stock solution of 4% (w/v) of DTZ was prepared using the same organic solvent mixture. An aliquot of 1 mL of DTZ stock solution was added to the lipid mixture. The organic solvents were then removed under vacuum in a rotary evaporator at 60 °C for 15 min leaving a thin film on the inside walls of the flask. The flask was left to rotate for another 1 h to ensure complete removal of traces of organic solvents. The film was then rehydrated by addition of 20 mL phosphate buffer saline (PBS), pH 7.4 and rotated for 30 min at 60 °C. The resulting niosomal suspension was left to mature over night at 4 °C and stored in a refrigerator for further studies.

**Table 1.** Composition, mean diameter and drug entrapment efficiency for DTZ-loaded niosomes.

	Molar ratio					
Formula	Span 60	Brij 52	CHOL	Mean particle Size ± SD^a^ (μm)	%EE (% ± SD)^a^	% Release efficiency ± SD^a^
F1	1	–	–	0.82 ± 0.12	54.77 ± 2.51	36.090 ± 0.219
F2	1	–	0.5	0.91 ± 0.14	55.52 ± 1.57	40.011 ± 0.303
F3	1	–	1	0.97 ± 0.13	66.26 ± 1.45	49.593 ± 0.395
F4	1	–	1.5	1.02 ± 0.18	40.44 ± 1.74	45.119 ± 0.401
F5	1	–	2	1.08 ± 0.17	38.18 ± 1.21	39.592 ± 0.353
F6	–	1	–	1.45 ± 0.15	20.58 ± 0.62	42.615 ± 0.366
F7	–	1	0.5	1.40 ± 0.16	25.89 ± 0.76	47.448 ± 0.379
F8	–	1	1	1.42 ± 0.20	29.92 ± 1.32	39.343 ± 0.395
F9	–	1	1.5	1.53 ± 0.14	33.40 ± 0.79	37.589 ± 0.371
F10	–	1	2	1.59 ± 0.11	35.17 ± 1.33	31.805 ± 0.213

^a^*n *=3

### Characterization of DTZ niosomes

#### Morphology and size

The shape and morphology of niosomes were characterized by transmission electron microscopy (TEM) (H-7500, Hitachi, Tokyo, Japan) working at an accelerating voltage of 80 kV. A drop of the prepared niosome was placed on a carbon-coated copper grid and left to adhere onto the carbon substrate for about 2 min before removal of sample in excess using a piece of filter paper. A drop of 2% (w/v) phosphotungstic acid was stratified onto the carbon grid and the excess staining agent was removed by a piece of filter paper. Finally the samples were air-dried and the thin film of stained niosomes was examined.

The average particle size and size distribution of each niosomal formula was determined by dynamic light scattering (DLS) analysis using Zetasizer ZEN3600 (Malvern Instruments Limited, Malvern, UK).

#### Determination of % entrapment efficiency

An accurately measured 0.5 mL of the prepared DTZ niosomes was centrifuged at 15.0 × *g*, 4 °C for 45 min. The isolated pellets were washed twice with PBS pH 7.4, vortexed and then centrifuged for another 45 min. The amount of entrapped drug was determined by lysis of the vesicles using 4 mL absolute methanol followed by sonication using a water bath sonicator (Soniclean 120 T, Transtek Systems PTY Ltd., Thebarton, Australia) for 15 min (Fang et al., 2001). The concentration of entrapped drug was determined by measuring the methanol solution obtained after sonication spectrophotometrically at a wavelength of 240 nm. The following equation was used to calculate the entrapment efficiency: $\%\mathrm{EE}=\frac{W_{L}}{W_{T}}\times 100$, where *W*_T_ is the total amount of the feeding drug and *W*_L_ is the total amount of loaded drug in niosomes.

### *In vitro* release studies

An accurately measured amount of niosomal suspension, equivalent to 480 μg of the drug, was placed in suitable dialysis bag (12 000–14 000 MW cutoff) and suspended in a beaker containing 60 mL PBS, pH 7.4, which acted as receptor compartment (Aggarwal & Kaur, 2005). The beaker was placed over a magnetic stirrer adjusted at 100 rpm and maintained at 37 ± 0.5 °C. At predetermined time intervals (0.25, 0.5, 0.75, 1, 2, 3, 4, 5 and 6 h), 3 mL samples were withdrawn and replaced with fresh buffer. The withdrawn samples were analyzed for drug content spectrophotometrically at 240 nm using PBS solution as a blank. The mean cumulative drug release percentage was plotted against time to study the mechanism of drug release and compare the release profiles of the different formulae.

The data was also used to determine the release efficiency (RE) obtained from the area under the release curve (AUC) at 6 h using the trapezoidal method. It is expressed as a percentage of the area of the rectangle corresponding to 100% release, for the same total time according to the following equation:

$$\mathrm{RE}=\frac{\int_{0}^{t}y\times \mathrm{dt}}{y\times t}\times 100$$

Where, *y* is the percentage of drug released at time *t.* Each *in vitro* release study was performed in triplicate and the release rate was determined from the slope of the line obtained on plotting cumulative amount of drug released versus time.

The mechanism of drug release from niosomes was determined by applying zero order, first order and second order kinetics and Higuchi diffusion model. The following linear regression equation were employed for zero order kinetics C*_t _=_ _*C*_0_−Kt* where C*_0_* is the zero time concentration of the drug, C*_t_* is the concentration of the drug at time *t* and *K* is the apparent release rate constant. First order kinetics was determined according to the equation,

*Ln* C*_t_= Ln* C*_0_−Kt*. For second order kinetics, the following equation was used: $\frac{1}{C_{t}}=\;\frac{1}{C_{o}}\;+\;\mathrm{Kt}$. Drug release following Higuchi model was determined using the equation *Q = Kt^1/2^*; where Q represents the fraction of drug released in time *t* and *K* is the Higuchi dissolution constant.

### *In vivo* assessment for DTZ-loaded niosomes

#### Animal protocol

Male Wistar rats (mean body weight of 200 ± 20 g) were selected for *in vivo* studies. Rats were housed into separate cages, fed a commercial laboratory diet with free access to water and fasted for 24 h prior to and during the pharmacokinetic study. All studies were carried out according to the guidelines of Ethics Committee on Animal Experiments, Faculty of Pharmacy, Cairo University, Egypt. The animals were divided into five groups (*n *= 6) marked as A, B, C, D and E. Groups A and B were treated nasally with one of two different DLT-loaded niosomal formulae that showed the highest *in vitro* RE. Group C was treated nasally with an aqueous solution of DTZ (400 μg/mL) in phosphate buffer pH 7.4 while groups D and E were nasally administered drug-free niosomal formulae used in A and B, respectively.

Formulation for the *in vivo* study was prepared as previously mentioned with a total drug concentration of 400 μg/mL. A dose of 200 μg of DLT per kg body weight (Dougherty et al., 1992), equivalent to 100 μL of the prepared formulas, was administered to rats of groups A, B and C. Rats were put in the supine position and 50 μL of each formula were instilled in each nostril using a micropipette. Rats of groups D and E were administered 50 μL in each nostril of drug free niosomes using the same technique.

#### Pharmacokinetic study on rats

After administration, 1 mL blood samples were collected from the orbital vein and placed in dried heparinized tubes after 0.5, 1, 2, 3, 4 and 6 h. Blood samples were centrifuged immediately at 3.0 × *g* for 10 min followed by separation of plasma into clean screw capped glass tubes that were stored at −80 °C till HPLC analyzed.

A reverse phase C-18 microbore column packed with ODS Hypersil (Thermo Fisher scientific, Waltham, MA) was used for HPLC analysis. The mobile phase was composed of an isocratic mixture of acetonitrile and 0.5% Triethanolamine in water (40:60 v/v) adjusted at pH 2.5 using orthophosphoric acid. The flow rate was adjusted to 1.3 mL/min. Liquid–liquid extraction was employed in extracting DLT from plasma samples using Verapamil HCl as the internal standard where 20 μL internal standard (25 μg/mL), and 2 mL ether were added to each 0.2 mL plasma sample. The mixture was then vortex mixed for 2 min followed by centrifugation at 3.0 × *g* for 10 min. The upper organic layer was separated and dried on a water bath at 40 °C and finally reconstituted with 130 μL mobile phase. Finally, 100 μL was injected onto the HPLC column and the eluent was monitored with a UV detector operating at 237 nm. The retention times for the drug and the internal standard were ∼3.7 and 5.2 min, respectively.

A calibration curve was constructed based on peak area ratio of the drug and internal standard by spiking known concentrations of the drug and internal standard into plain plasma, being extracted and analyzed by the same above-mentioned procedure.

Samples were quantified using peak area ratio of the drug over the internal standard. The recovery of the extraction procedure for DLT was calculated by comparing the peak area ratio obtained after extraction with that of aqueous solutions of corresponding concentrations without extraction. The accuracy was expressed as percentage error, obtained by calculating the percentage of difference between the measured and the spiked concentration over that of the solution value.

Non-compartmental analysis was performed by using WinNonlin® software (Pharsight Co., Mountain View, CA). The area under the plasma concentration–time curve (AUC) was calculated using the linear trapezoidal method. The peak plasma concentration (C_max_), the time to reach the peak plasma concentration (*T*_max_), the time to reach half the plasma concentration (*t*_1/2_), elimination rate constant (*K*_e_), and the mean residence time (MRT) were determined.

### Statistical analysis

Data Analysis was performed using the statistical package for sciences, SPSS version 16.0 (SPSS Inc., Chicago, IL). Intergroup differences were assessed using the paired *t*-test for DTZ concentration and the ANOVA test for the physicochemical characterization and *in vitro* releases studies. The level of statistical significance was set at *p *< 0.05.

## Results and discussion

### Preparation and characterization of niosomes

Our main goal was to formulate DTZ niosomes for nasal delivery as an alternative approach to optimize its systemic bioavailability. To prepare DTZ loaded niosomal vesicles, we used thin-film hydration method which is the most common technique for niosomes preparation. This method has been previously used for formulation of niosomes entrapping both large and small molecular weight therapeutic agents (Moghassemi & Hadjizadeh, 2014). The effect of formulation parameters such as the type of surfactant and the surfactant/lipid molar ratio on the physicochemical properties and *in vitro* release of DTZ from niosomes were investigated in order to select an optimum formulation for *in vivo* studies.

**Figure 1.**
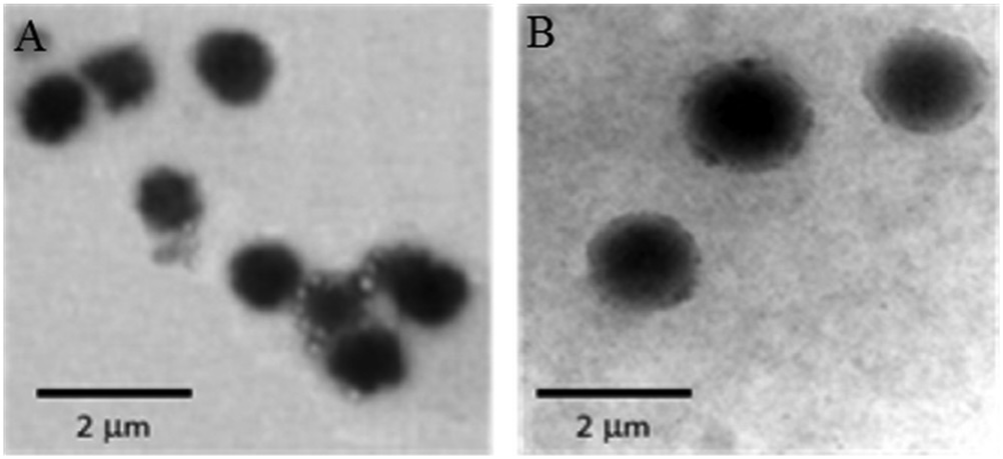
TEM micrographs of selected niosomes prepared from: A) Span 60 and B) Brij-52 at 20 000× magnification.

The determination of morphological pattern of the prepared niosomes using TEM mostly showed formation of unilamellar spherical niosomes with definite margins and aqueous core (Figure 1) with few aggregations of discrete vesicles occasionally observed.

Previous studies suggested that the size of the niosomes is dependent on many factors including the degree of hydration of the hydrophilic head, the hydrophobic character of the surface active agent, the properties of the molecules in the bilayer, distance between the bilayers and the number of bilayers present (Hao et al., 2002; Manosroi et al., 2003; Balakrishnan et al., 2009). The mean particle size of the niosomes containing different ratios of Span 60/CHOL (F1–F5) was in the range of 0.82–1.08 μm with PDI ranging from 0.07 to 0.26. Niosomes composed of Brij 52/CHOL (F6–F10) had particle size mean values in the range of 1.45–1.59 μm with PDI ranging from 0.03 to 0.37 for niosomes. The obtained mean sizes of the vesicles were in good agreement with those observed in TEM micrographs. The relatively larger particle size of the Brij 52 niosomes compared to those containing Span 60 may be attributed to the higher HLB value of Brij 52 (5.3) which reflects higher contribution of its hydrophilic head that is well hydrated with water. On the contrary, the use of more hydrophobic surfactant such as Span 60 with low HLB value (4.7) and surface free energy results in formation of smaller size vesicles.

The addition of CHOL is essential for the formation of niosomes as it significantly affects a number of membrane properties such as stability, ion permeability, elasticity, fluidity, aggregation, size and shape. The presence of CHOL increases the rigidity of the bilayer by reducing the phase transition temperature peak of the vesicles and increases the chain order of liquid state bilayers as well (Kazi et al., 2010; Essa, 2010). As shown in Table 1, the increase in molar fraction of CHOL generally resulted in an increase in vesicle size. This may be due to disturbance imparted in the vesicular membrane by increased hydrophobicity in presence of higher CHOL content and thereby formation of larger vesicles with more thermodynamic stability (Essa, 2010).

### Entrapment efficiency

Niosomal vesicles containing Span 60 showed EE% ranging from 38 to 66%, whereas niosomes prepared using Brij 52 had EE% in the range of 20 to 35% only (Table 1). The results were in good correlation with the particle size measurements where the increase in size of niosomes containing was accompanied by an increase in EE%.

The entrapment efficiency of Span 60 based niosomes was superior to their corresponding Brij 52 based niosomes. This can be explained by the fact that Span 60 has a higher phase transition temperature (53 °C) than Brij 52 (32.5 °C) which in turn decreases the fluidity and leakage of bilayer. In addition, Span 60 exhibits lower HLB value (4.7) with a longer C17 chain compared to Brij 52 (HLB 5.3 and C16), which sequentially makes it more hydrophobic leading to better holding hydrophilic drugs inside its core (Yoshioka et al., 1994).

Clearly EE% of DTZ was affected by the surfactant/CHOL ratio as in Brij 52 niosomes EE% increased by ∼1.5 times with increasing CHOL molar ratio from zero (F6) to 2 (F10) showing a linear relationship between CHOL molar ratio and EE% of DTZ niosomes prepared with Brij 52. These results can be explained by the fact that an increase in CHOL content is usually associated with an increase of the micro viscosity of the membrane indicating more rigidity of the bilayers. The bilayer hydrophobicity as well as stability increased with increasing CHOL content leading to decreasing permeability, which may have led to efficiently trapping DTZ inside the niosomes (Bernsdorff et al., 1997).

EE% in Span 60 niosomes increased from 54 to 66% with increasing CHOL molar ratio from zero to 1 in F1 to F3, respectively. However, further increase CHOL molar fraction was accompanied by a decrease in EE% (F4 and F5). Previous studies showed that increasing CHOL beyond a certain optimum concentration may result in a reduction or no effect in EE% of hydrophilic drugs which may be due to disruption in the physical organizational structure of the bilayer leading to leakage (Chaw & Ah Kim, 2012; Kamboj et al., 2014).

### *In vitro* release profile

**Figure 2.**
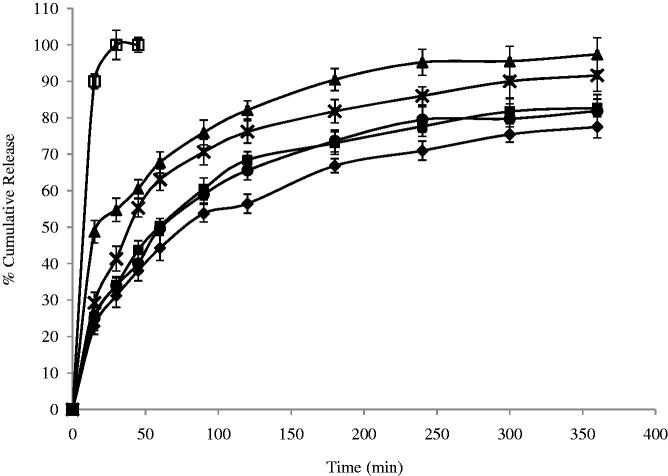
Effect of surfactant/CHOL ratio on *in vitro* cumulative release of DTZ from span 60 niosomes (♦) F1; (▪) F2; (▴) F3; (ȕ) F4; (•) F5 and (□) free drug. Data are mean values ± standard deviation (*n *= 3).

**Figure 3.**
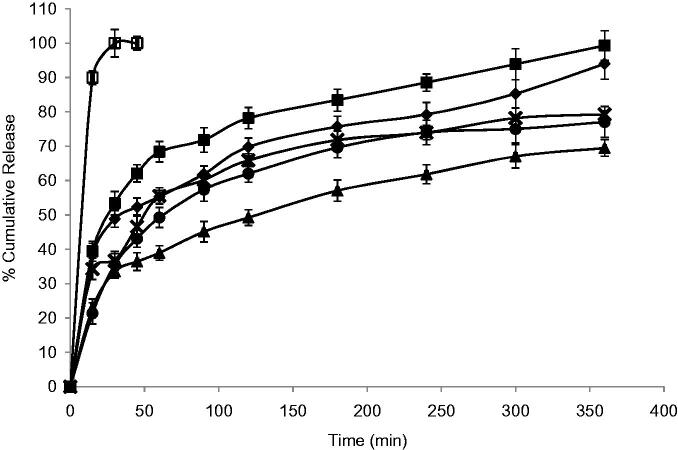
Effect of surfactant/CHOL ratio on *in vitro* cumulative release of DTZ from Brij-52 niosomes (♦) F6; (▪) F7; (▴) F8; (ȕ) F9; (•) F10 and (□) free drug. Data are mean values ± standard deviation (*n* = 3).

The release profile of DTZ from all niosomal formulae exhibited a biphasic pattern (Figures 2 and 3) with an initial rapid drug release of about 49–82% of the incorporated drug observed during the first 2 h, followed by a slower release pattern in the next 4 h in which only further 14–20% of DTZ were released. This could be generally attributed to the initial release of the free drug in the niosomal suspension followed by release of the encapsulated drug inside the niosomes. The highest percentage release efficiencies values were observed for formulas F3 and F7 which showed 49.59 and 47.45%, respectively (Table 1). The fitting of the release profile data to different order kinetic equations showed that DTZ release from all formulae followed Higuchi order release kinetics with R^2^ values ranging between 0.91 and 0.99. This result is generally in agreement with previous studies that reported that drug-loaded niosomes provide a controlled release pattern following Higuchi’s square root model (Chougule et al., 2007; Ruckmani & Sankar, 2010).

Niosomes, being an example of nanocarrier systems, can be regarded as nano drug depots capable of controlling and prolonging drug release (Siegel & Rathbone, 2012). The %RE and mechanism of release of DTZ from the prepared niosomes were affected by both the type of surfactant and amount of CHOL in the lipid bilayer. Niosomal formulation F1 and F2, containing Span 60, showed significantly lower RE (*p *< 0.05) when compared to their corresponding Brij 52 niosomal formulations, F6 and F7, with similar surfactant/CHOL ratios. Brij 52 niosomes, being more hydrophilic, can have greater interaction with surrounding aqueous release media which may suggest faster drug diffusion and release. Increasing the amount of CHOL in the lipid bilayer F3–F5 resulted in a significantly higher (*p *< 0.05) RE than their corresponding formulations F8–F10, respectively. The increase in the amount of incorporated CHOL into the niosomal formulation is accompanied by an increase in their lipophilicity. Having an amphiphilic molecule bearing both highly hydrophobic group and highly hydrophilic group, as in the case of Brij 52 niosomes, can form rigid membrane with good barrier function against aqueous compounds in presence of increasing amount of CHOL (Manosroi et al., 2003).

The mechanism of drug release from niosomes is dominated by the partitioning of the drug and rigidity of the niosomal membrane (Ruckmani & Sankar, 2010). DTZ with a pKa 7.5 and log P octanol/water = 2.79, being present in pH 7.4 triggers the presence of the unionized form which in turn would diffuse through the niosomal membrane easing the release of the drug from the niosomal formulation. This could be the rationale behind the significantly higher (*p *< 0.05) RE from F3 containing equal molar concentration of span 60 and CHOL. Both the highly lipophilic span 60 and the high CHOL concentration may enhance the partitioning and diffusion of the unionized form of the drug through the lipophilic niosomal membrane (Bernsdorff et al., 1997). On the other hand, with more hydrophilic surfactant such as Brij 52, optimum RE was observed in F7 with lower CHOL content (Brij 52: CHOL 1:0.5) and less rigid lipid bilayer decreasing its ability to hold the entrapped drug inside its core (Bernsdorff et al., 1997).

### Pharmacokinetic study on male Wistar rats

The nasal route was used in this study for administration of DTZ-loaded niosomes to substitute per oral route to improve the bioavailability of the drug and avoid its extensive metabolism by first pass effect. Other alternative for efficient delivery of DTZ have also been studied including mucoadhesive discs for buccal delivery (Haider et al., 2014) and polymeric matrices with or without iontophoresis for transdermal delivery (Parhi & Suresh, 2016; Mundada & Avari, 2011). *In vivo* studies using both approaches showed significant enhancement in bioavailability of DTZ but were limited by a lengthy *T*_max_ (>5 h). This may be due to slow rate of drug absorption from buccal mucosa and skin or slow rate of drug release from the studied formulae.

**Figure 4.**
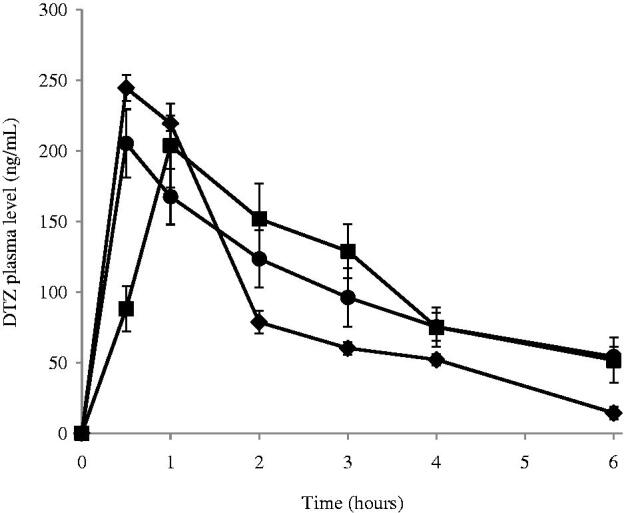
Plasma concentration following the administration of (•) F3; (▪) F7 and (♦) control drug solution by intranasal route at the amount of 250 μg/Kg of DTZ in male Wistar rats. Blood was collected before administration at t tm0 and after administration at t = 30 min, 1, 2, 3, 4 and 6 h. Points represent the mean ± standard deviation for *n *=6 per group.

Based on the results of the *in vitro* release studies, F3 has shown both maximum EE% and %RE among all formulae while F7 showed the best RE% among the Brij containing niosomes. Accordingly, both F3 and F7 were selected for further *in vivo* pharmacokinetic studies in male Wistar rats as a common model for nasal administration (Mei et al., 2008; Na et al., 2010). There was no sign of inflammation or swelling in the nasal cavity of the rats after administration of niosomal formulae. The mean plasma levels of DTZ for the two chosen formulae and the control group are represented in Figure 4 while the computed pharmacokinetic parameters for the three groups are shown in Table 2. The time of peak plasma levels *T*_max_ for both treatments (groups A and B) was 1 h or less which shows the significance of nasal delivery compared to other routes of administration. Statistical analysis of the measured pharmacokinetic parameters showed a significant increase in the value of *T*_max_ in group B; treated nasally with formula F7 (*p* < 0.0001) when compared to group A; nasally treated with F3 which illustrate the effect of niosomes composition on the rate of drug absorption. This could be attributed to the fact that amphiphilic molecules bearing both highly hydrophobic group and highly hydrophilic group F7, can form a rigid membrane with good barrier function against hydrophilic therapeutic agents which would delay their release from the niosomes leading to a longer *T*_max_ (Manosroi et al., 2003) which is in accordance with the results obtained from the *in vitro* release study. The decrease in half-life of elimination in group B may be attributed to lower EE% in F7 which leads to faster clearance of the drug. Both F3 and F7 exhibited a significantly higher *t*_1/2_, MRT, AUMC_0–∞_ and AUC_0–∞_ than the control group (*p* < 0.0001) showing that niosomes increased the extent of drug absorption. In addition, the area under the first moment curve AUMC_0–6_ (*p *=0.0004) and AUC_0–6_ (*p *=0.0005) of the nasal niosomal formulae; groups A and B, were significantly higher than the control group. The elimination rates constant K_e_ as well as the ratio C_max_/AUC_0–∞_ were significantly (*p* < 0.0001) lower in both F3 and F7 compared to the control group. These results comply with the expected attitude of niosomal formulations. Niosomes, being an example of nano carrier systems, can be regarded as nano drug depots controlling and prolonging drug release. By protecting the drug inside its core, niosomes could extend the MRT as well as increasing the half-life of DTZ, consequently increasing its AUC and AUMC (Siegel & Rathbone, 2012).

**Table 2.** Pharmacokinetic parameters of DTZ in rats of groups A, B and C.

Parameter	Group A	Group B	Group C
C_max_ (ng/mL)	220.08 ± 10.2	203.81 ± 12.07	244.66 ± 3.75
*T*_max_ (h)	0.50 ± 0.08	1.00 ± 0	1.00 ± 0
MRT (h)	4.60 ± 0.57	4.54 ± 0.31	2.22 ± 0.07
Half Life (h)	3.06 ± 0.40	2.81 ± 0.21	1.46 ± 0.08
AUC _0–6h_ (ng.h.mL^−1^)	615.46 ± 38.25	642.14 ± 32.22	518.29 ± 6.37
AUC _0–∞_ (ng.h.mL^−1^)	862.26 ± 95.42	876.41 ± 27.59	550.06 ± 6.87
AUMC _0–6h_ (ng.h^2^.mL^−1^)	1490 ± 38.25	1569.79 ± 32.22	1034.69 ± 6.37
AUMC _0–∞_ (ng.h^2^.mL^−1^)	4229.71 ± 864.02	3989.15 ± 313.58	1334.45 ± 97.86
Ke (h^−1^)	0.25 ± 0.04	0.25 ± 0.02	0.48 ± 0.02
Cmax/AUC _0–∞_ (h^−1^)	0.28 ± 0.04	0.23 ± 0.012	0.45 ± 0.01

## Conclusion

In this study, DTZ-loaded niosomes were successfully prepared using different combinations of various nonionic surfactants and CHOL. The physicochemical properties and *in vitro* release of DTZ from niosomes were affected by the type of nonionic surfactant and the surfactant-to-CHOL molar ratio. The developed niosomes improved the pharmacokinetic parameters of DTZ and boosted its bioavailability through prolonging its duration of action inside the body as well as decreasing its elimination rate constant compared to the free drug (control group) showing the potential of niosomes as promising nanoparticulate carriers for nasal delivery DTZ.
